# Preliminary Evidence for IL-10-Induced ACE2 mRNA Expression in Lung-Derived and Endothelial Cells: Implications for SARS-Cov-2 ARDS Pathogenesis

**DOI:** 10.3389/fimmu.2021.718136

**Published:** 2021-09-27

**Authors:** Adriana Albini, Luana Calabrone, Valentina Carlini, Nadia Benedetto, Michele Lombardo, Antonino Bruno, Douglas M. Noonan

**Affiliations:** ^1^ Laboratory of Vascular Biology and Angiogenesis, Istituti di Ricovero e Cura a Carattere Scientifico (IRCCS) MultiMedica, Milan, Italy; ^2^ Unit of Molecular Pathology, Biochemistry and Immunology, IRCCS MultiMedica, Milan, Italy; ^3^ Department of Cardiology, IRCCS MultiMedica, Milan, Italy; ^4^ Laboratory of Innate Immunity, Unit of Molecular Pathology, Biochemistry and Immunology, IRCCS MultiMedica, Milan, Italy; ^5^ Immunology and General Pathology Laboratory, Department of Biotechnology and Life Sciences, University of Insubria, Varese, Italy

**Keywords:** ACE2, IL-10, COVID-19 pro-inflammatory cytokines, SARS-COV-2, cytokine storm, metformin, sartans, ARDS

## Abstract

Angiotensin-converting enzyme 2 (ACE2) is a receptor for the spike protein of SARS-COV-2 that allows viral binding and entry and is expressed on the surface of several pulmonary and non-pulmonary cell types, with induction of a “cytokine storm” upon binding. Other cell types present the receptor and can be infected, including cardiac, renal, intestinal, and endothelial cells. High ACE2 levels protect from inflammation. Despite the relevance of ACE2 levels in COVID-19 pathogenesis, experimental studies to comprehensively address the question of ACE2 regulations are still limited. A relevant observation from the clinic is that, besides the pro-inflammatory cytokines, such as IL-6 and IL-1β, the anti-inflammatory cytokine IL-10 is also elevated in worse prognosis patients. This could represent somehow a “danger signal”, an alarmin from the host organism, given the immuno-regulatory properties of the cytokine. Here, we investigated whether IL-10 could increase ACE2 expression in the lung-derived Calu-3 cell line. We provided preliminary evidence of ACE2 mRNA increase in cells of lung origin *in vitro*, following IL-10 treatment. Endothelial cell infection by SARS-COV-2 is associated with vasculitis, thromboembolism, and disseminated intravascular coagulation. We confirmed ACE2 expression enhancement by IL-10 treatment also on endothelial cells. The sartans (olmesartan and losartan) showed non-statistically significant ACE2 modulation in Calu-3 and endothelial cells, as compared to untreated control cells. We observed that the antidiabetic biguanide metformin, a putative anti-inflammatory agent, also upregulates ACE2 expression in Calu-3 and endothelial cells. We hypothesized that IL-10 could be a danger signal, and its elevation could possibly represent a feedback mechanism fighting inflammation. Although further confirmatory studies are required, inducing IL-10 upregulation could be clinically relevant in COVID-19-associated acute respiratory distress syndrome (ARDS) and vasculitis, by reinforcing ACE2 levels.

**Graphical Abstract:**
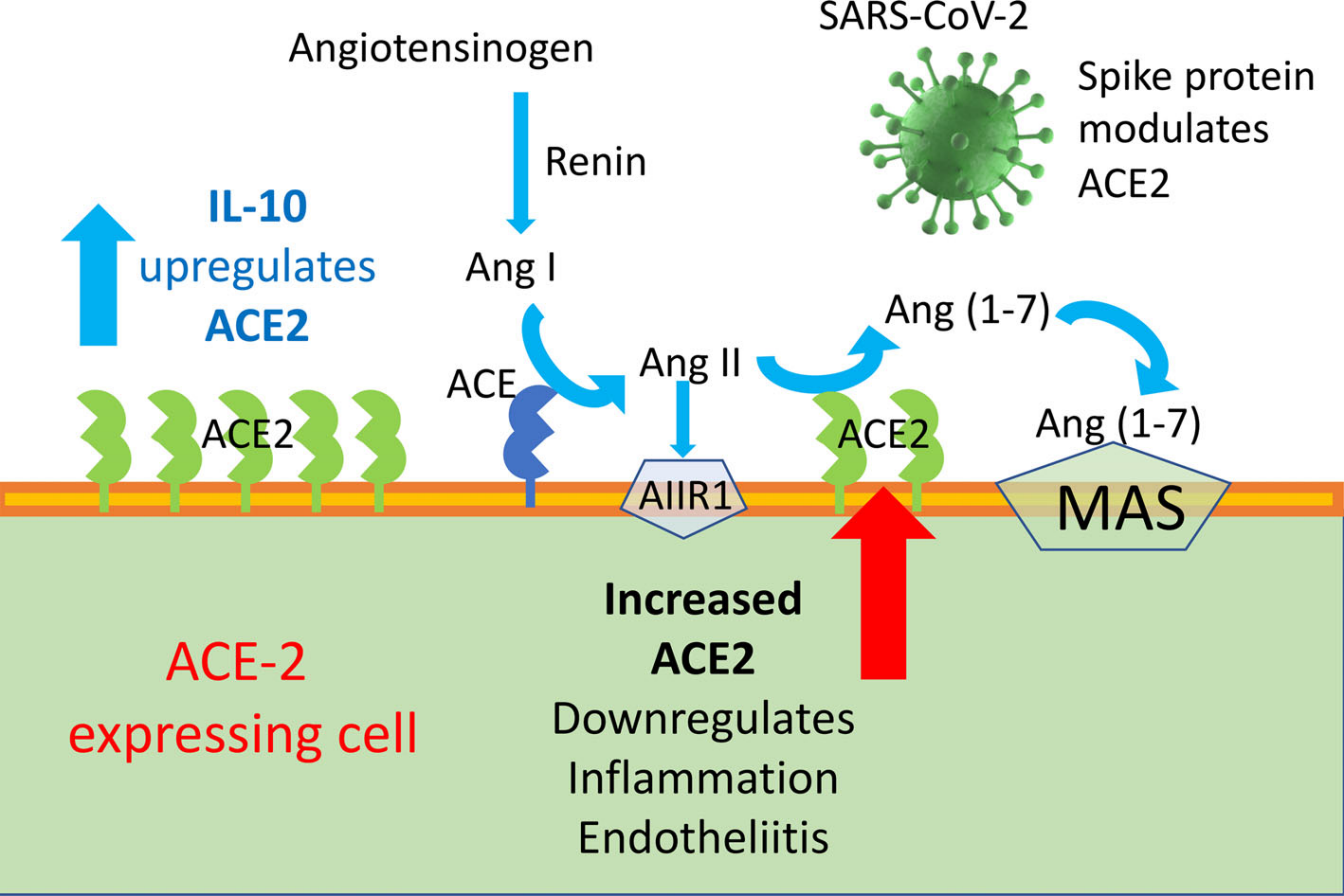
ACE2 enzyme is a negative regulator of RAAS providing a crucial link between immunity, inflammation, increased coagulopathy, and cardiovascular disease, thereby serving as a protective mechanism against inflammation, heart failure, myocardial infarction, lung disease, hypertension, vascular permeability, and diabetes. ACE2 function, following SARS-CoV binding, is reduced due to endocytosis and proteolytic cleavage and there are high levels of Ang II in the blood of patients with COVID-19. Consequently, the upregulation of human ACE2 induced by IL-10 in SARS-CoV-2-infected patients could be clinically useful, due to the protection elicited by the increased activity of ACE2 and possibly angiotensin(1–7).

## Introduction

Coronaviruses are animal viruses, but they mutate towards strains that can infect directly humans (1, 2). A novel strain, 2019-nCoV or COVID-19, giving rise to SARS-CoV-2, has emerged at the end of 2019 (1, 3). The understanding of COVID-19 pathogenesis and the occurrence of high COVID-19-associated death burden became an urgency within the pandemic. Thousands of papers have been published, on epidemiology, diagnosis, and cure. Vaccination appears to be the most successful strategy against COVID-19 so far; however, numerous pharmacological approaches are being studied.

In this scenario, challenging open questions for immunologists remain partially unresolved.

Coronaviruses easily replicate in epithelial cells of the respiratory tract, but not exclusively: there are a large number of cells that can harbor the virus (4). Epithelial cell infection leads to cytopathic changes of the mucosae, mostly respiratory and enteric. Endothelial cells might be involved in the arterial as well venous thrombotic events (4). Within the respiratory disease, which can be mild or severe, human coronavirus infections can induce inflammation and immune suppression with respiratory tract infection and organ dysfunction, until acute respiratory distress syndrome (ARDS) that can be lethal (3, 5, 6).

Virus pathogenesis is frequently associated with the intensified production of proinflammatory cytokines in some patients and was evident with the previous SARS (7). Proinflammatory cytokines and chemokines act as the necessary early immune response against pathogens. However, an increased and non-controlled immune response has been associated with the high virulence of SARS-CoV (8, 9), indicated as “cytokine storm”. Peripheral blood and lungs of SARS patients were characterized by elevated levels of proinflammatory cytokines, such as IL-1, IL-2, IL-6, IL-8/CXCL8, and chemokines, including CXCL10 and MCP-1/CCL2, which were associated with disease severity (10–14).

SARS-CoV infects type 2 pneumocytes and ciliated bronchial epithelial cells engaging angiotensin converting enzyme 2 (ACE2) as a receptor, which processes angiotensin II (Ang II) to Ang (1–7). The novel SARS-CoV-2 virus employs ACE2 as a receptor, similar to SARS-CoV (15–18). ACE2 is a membrane-bound peptidase expressed in lungs, heart, kidneys, and intestine as well as arterial and venous endothelial cells in all organs studied (4, 19). Ang II is a key effector of the renin–angiotensin–aldosterone system (RAAS) and exerts its biological functions through type 1 and 2 receptors (AT1R and AT2R) (20). RAAS has a key role in blood pressure homeostasis and electrolyte balance, while Ang II through AT2R is also involved in the development of inflammatory reactions and in the control of the kinin–kallikrein system, with important implications for the coagulation cascade. SARS-CoV infection decreases the expression of ACE2 (21), with possible relevance for the development of lung fibrosis reported in SARS patients months after their clearance of the viral infection (22–26); in fact, ACE2 exerts an anti-inflammatory role.

COVID-19 patients in particular display elevated levels of IL-6, and this is the cytokine so far mostly studied, with investigations also on the possible therapeutic use of anti-IL-6 agents, such as tocilizumab (27). However, as reported above, other cytokines are present in altered levels.

Increasing evidence supports the involvement of IL-10, although few investigations have been addressed to this molecule. IL-10, the main member of the IL-10 superfamily, plays a critical role in the resolution of peripheral inflammation, thus being largely investigated as anti-inflammatory cytokine (28, 29). Since its initial discovery, it has been found that IL-10 is produced by different leukocytic cell types, such as monocytes, granulocytes, and non-immune cells, including epithelial cells and keratinocytes (30, 31). Despite the numerous published papers, the ultimate role of IL-10 in disease has not been fully determined, due to the extremely heterogenous immunological contexts that regulate its functions and the documented controversial effects (32–34). *In vivo*, IL-10 exerts dual effects on NK activities, with some reports documenting increased cytolytic activities and others showing an opposite effect (32, 35). The tissue microenvironment or *in vitro* surroundings plays a role in IL-10 inhibition or activation of immune cells (32).

In severe/critically ill patients, a dramatic increase of interleukin IL-10 has been described as a crucial feature of COVID-19. COVID-19 patients in the intensive care unit (ICU) have been reported to exhibit increased systemic levels of IL-10, as compared to non-ICU patients (3, 36, 37). Elevated levels of IL-10 have strong correlations with those of IL-6 and C-reactive protein (38). This clearly suggests the presence of IL-10 in COVID-19 severity.

Patients with severe and fatal disease had significantly increased IL-6 and IL-10. These were strong discriminators in Chinese cohorts (39) and Brazilian cohorts (40). The levels of IL-10 are positively correlated with CRP amount (38). Elevation of IL-6, IL-10, and C-reactive protein is a reliable indicator of severe COVID-19 (41). IL-10 was elevated in severe but not mild cases after the virus infection; IL-10 at week 1 may predict patient outcomes (42). IL-10 and IL-10/lymphocyte count at emergency department presentation were described as independent predictors of COVID-19 severity (43). Moreover, elevated IL-10 was more strongly associated with outcomes than pro-inflammatory IL-6 or IL-8. IL-6 and IL-10 are found to predict disease severity (38). Elevated serum levels of IL-6, IL-10, and tumor necrosis factor alpha (TNFα) in non-survivors have been detected, compared to those in the survivors (44). Also, a fatal outcome has been observed in patients with severe COVID-19 with kinetic variations in IL-6, IL-8, and IL-10, independently of sex, age, absolute lymphocyte count, direct bilirubin, hypertension, and chronic obstructive pulmonary disease (44). Two meta-analyses on IL-6 and IL-10 circulating levels found a correlation between the disease severity and mortality in COVID-19 patients (45, 46). Since IL-10 is reported as an anti-inflammatory cytokine, elevated levels could represent a reaction of the organism to curb inflammation, a sort of alarmin-like signal (47).

We hypothesize that IL-10 could be a danger signal, and its elevation could possibly represent a negative feedback mechanism suppressing inflammation.

Although spike-ACE2 binding in pneumocytes, as well as other affected cells, is required for viral entry, paradoxically, treatments that increase ACE2 may be beneficial in mitigating the complications of COVID-19, by curbing inflammation. Therefore, ACE2 expression seems to be a two-faced Janus for the development of human COVID-19 disease severity. In the RAAS, Angiotensin converting enzyme 2 (ACE2)–Ang 1-7–Mas represents a protective arm to contrast the deleterious effect of ACE1–Ang II, which induces systemic and pulmonary hypertension. ACE2 activates anti-inflammatory pathways after tissue injury (48). SARS-CoV-1 was found to decrease ACE2 expression after binding, and low levels of ACE2 have been implicated in cardiovascular impairments and ARDS (20, 49).

Anti-inflammatory drugs may offer cardiopulmonary protection in COVID-19 *via* enhanced ACE2 expression. The paradoxical effect of stimulating SARS-COV-2 receptor ACE2 messenger expression could lead to both enhanced entry route in the cells and, at the same time, reduced inflammatory cytokine production and protection from further damage.

In this Brief Research Report, we show our preliminary data on a possible role for IL-10 in trying to mitigate COVID-19 pathogenesis, by enhancing ACE2 expression in lung cells (see Graphical Abstract). We show that treatment with IL-10 of Calu-3, cells of lung epithelial origin, enhances mRNA expression of ACE2, in a dose-dependent manner, as measured by quantitative qPCR. Given the involvement of endothelial cells in COVID-19 pathogenesis, we also stimulated cultured human endothelial cells to see if a similar scenario as in Calu-3 cells might occur. We found that ACE2 is upregulated by IL-10 also on human umbilical vein endothelial cells (HUVECs). Expression of ACE2 on Calu3 and HUVECs was also detected by Western blot, to confirm it at the protein level.

Treatment with biguanide metformin, based on some evidence of beneficial effects in SARS-CoV-2 (50, 51), was also tested, and was found to upregulate ACE2 in lung and HUVECs. Sartans, regulators of the RAAS system, did not induce increase or reduction of ACE2 messenger to a significant extent.

We point that our preliminary data provide a hint to the mechanism of a potential role of IL-10 in SARS-CoV-2-associated disease severity and ARDS, by elevating ACE2 expression, and that IL-10 stimulation could have therapeutic potential and therefore we propose our data here as a “rapid preliminary report”.

## Materials and Methods

### Cytokines, Angiotensin II Receptor and ACE inhibitors, and Metformin

IL-1β and IL-10 cytokines were purchased from Miltenyi Biotec (Bergisch Gladbach, Germany) and resuspended in sterile water. TNFα was purchased from Peprotech (Rocky Hill, NJ, USA) and resuspended in sterile water. Olmesartan, losartan, and enalapril were purchased from Sigma-Aldrich and dissolved in sterile water. Metformin was purchased by Sigma-Aldrich and dissolved in sterile water.

### Cell Line Culture and Maintenance

Calu-3 cells were purchased by American Type Culture Collection (ATCC) and cultured in RPMI 1640 medium (Euroclone, Pero, MI, Italy) supplemented with 10% of FBS, 2 mM l-glutamine (Euroclone), 100 U/ml penicillin, and 100 μg/ml streptomycin (Euroclone).

HUVECs were purchased by ATCC and cultured in endothelial cell basal medium (EBM, Lonza), supplemented with endothelial cell growth medium (EGM™ SingleQuots™, Lonza), 10% of FBS, 2 mM l-glutamine (Euroclone), 100 U/ml penicillin, and 100 μg/ml streptomycin (Euroclone) and used between three and five passages. Clau3 and HUVECs were regularly tested for absence of mycoplasma contamination.

### Cell Treatments

Calu-3 cells (1 × 10^6^ cells/dish) were seeded in 100-mm Petri dishes in RPMI 1640 medium (Euroclone, Pero, MI, Italy) supplemented with 10% of FBS, 2 mM l-glutamine (Euroclone), 100 U/ml penicillin, and 100 μg/ml streptomycin (Euroclone). Following cell adhesion, fresh complete medium with IL-10 (1, 5, and 25 ng/ml), TNFα (50 ng/ml), IL-1β (25 ng/ml), olmesartan (10 µM), losartan (10 µM), and metformin (10 mM) were added for 24 h at 37°C.

HUVECs (1 × 10^6^ cells/dish) were seeded in 100-mm Petri dishes in endothelial cell basal medium, 10% of FBS, 2 mM l-glutamine, 100 U/ml penicillin, and 100 μg/ml streptomycin. Following cell adhesion, fresh complete medium with IL-10 (1, 5, and 25 ng/ml), TNFα (50 ng/ml), IL-1β (25 ng/ml), olmesartan (10 µM), enalapril (10 µM), and metformin (10 mM) were added for 24 h at 37°C. Following treatments, cells were used for further analysis by qPCR, Western blot, and flow cytometry.

### Real-Time PCR

ACE2 primers for qPCR and the housekeeping β-actin (**Supplementary Table S1**) were designed using the NCBI Primer-BLAST tool and purchased from Integrated DNA Technologies (IDT, Coralville, IA, USA). Total RNA was extracted using TRIzol method, following separation with chloroform precipitation of RNA with isopropanol (Sigma-Aldrich). RNA pellet was washed twice with 75% ethanol (Sigma-Aldrich) and resuspended in nuclease-free water. RNA concentration was determined using Nanodrop Spectrophotometer ND-1000 (Thermo Fisher Scientific). Reverse transcription was performed using the SuperScript VILO cDNA synthesis kit (Thermo Fisher Scientific), starting from 1,000 ng of total RNA. Quantitative real-time qPCR was performed using SYBR Green Master Mix (Applied Biosystems) on QuantStudio 6 Flex Real-Time PCR System Software (Applied Biosystems). All reactions were performed in duplicate, and the experiment was repeated four times. The relative gene expression was expressed relative to non-treated cells normalized to the housekeeping gene. Cycles up to 35 were taken into account.

### Western Blotting Analysis

Cell lysates were obtained using RIPA buffer, supplemented with protease and phosphatase inhibitor cocktails (Roche Diagnostics GmbH). Proteins (30 µg) were loaded on NuPAGE Novex 4%–12% Bis-Tris Gel (Life Technologies) and transferred to a PVDF membrane Amersham Hybond (GE Healthcare Bio-Sciences, Pittsburgh, PA, USA). Membranes were incubated overnight at 4°C with the primary antibodies ACE2 (Invitrogen, dilution 1:1,000). Then, membranes were washed three times in TBS with 0.05% Tween 20 (TBS-T, pH 7.4), and incubated for 1 h at room temperature, with peroxidase-linked anti-rabbit IgG secondary antibody (GE Healthcare Bio-Sciences, Pittsburgh, PA, USA, dilution 1:2,000). Specific protein bands were detected by acquisition with the Alliance Q9 Atom System (Uvitec, Cambridge, UK). Protein expressions were normalized to β-actin (Cell Signaling Technology, dilution 1:1,000). Western blot data were analyzed using Q9 Alliance software (Uvitec, Cambridge, UK) to determine the band optical density (OD).

### Statistical Analysis

Statistical analysis was performed using the GraphPad Prism software, v9. Results are shown as mean ± SEM, one-way or two-way ANOVA, followed by Tukey’s post-hoc test. *p* values ≤ 0.05 were considered statistically significant.

## Results

### IL-10 Treatment of Calu 3 (Lung) Cells

**Figure 1:**
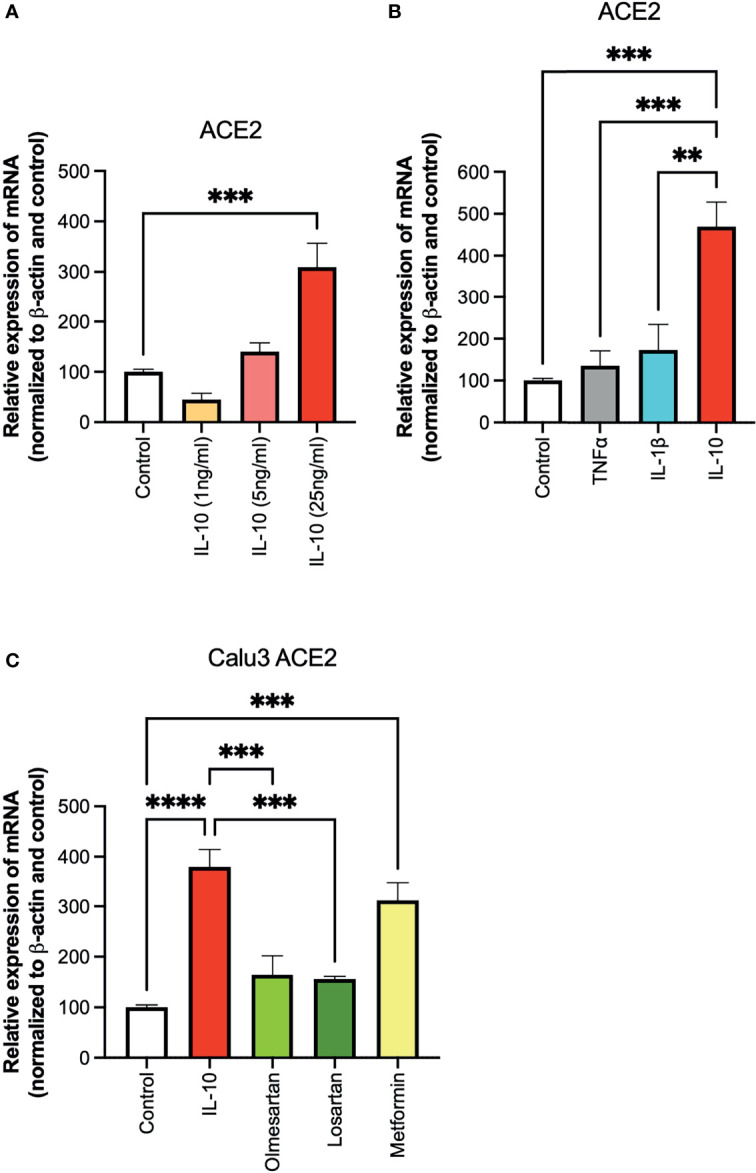
Effects of IL-10 on ACE2 expression in Calu-3 cells by qPCR. **(A)** The ability of IL-10 (1 to 10 ng/ml) to induce ACE2 expression in Calu-3 cells was determined, following 24 h of stimulation, by qPCR. IL-10 increased ACE2 expression in a dose-dependent manner; *N* = 5–7. **(B)** The effects of IL-10 (25 ng/ml) to induce ACE2 expression in Calu-3 cells were compared to the pro-inflammatory cytokines TNFα (50 ng/ml) and IL-1β (25 ng/ml), following 24 h of treatment; *N* = 5. IL-10 (25 ng/ml) increased ACE2 expression as compared to TNFα (50 ng/ml), IL-1β (25 ng/ml), and control untreated cells. **(C)** The effects of IL-10 (25 ng/ml) on ACE2 expression in Calu-3 was determined by qPCR, as compared to olmesartan (10 µM) or losartan (10 µM); olmesartan (10 µM) or metformin, (10 mM), by qPCR. *N* = 5. Non-significant effects was seen with sartans but metformin (10 mM) increased ACE2 expression. Data are shown as mRNA relative expression, normalized to β-actin and control, mean ± SEM, one-way ANOVA, ^**^ *p* < 0.01, ^***^ *p* < 0.001, Control: control vehicle cells.

To investigate the effect of IL-10 on ACE2 expression on Calu 3 cells at the transcriptional level, we performed quantitative real-time PCR (qPCR) analysis on cells treated with different doses of IL-10. ACE2 was found to be differentially expressed in IL-10-treated cells as compared to non-treated ones, in a statistically significant manner by using 25 ng/ml dosage (**Figure 1A**). We also compared the effects of the inflammatory cytokines TNF-α and IL-1β, at the same concentration, without observing an increase in ACE2 expression (**Figure 1B**). The ACE2 levels in Calu3 treated with IL-10 (25 ng/ml) were statistically significantly higher than in control, TNF-α, and IL-1β in Calu 3 cells.

We also tested inhibitors of the ACE system, and a non-related anti-diabetic, anti-inflammatory drug. No statistically significant difference in ACE2 expression was detected in Calu3 cells treated with the olmesartan and losartan (angiotensin II receptor inhibitors), as compared to control untreated cells (**Figure 1C**). Cells treated with the biguanide metformin exhibited statistically significantly upregulation of ACE2 (**Figure 1C**).

**Figure 2:**
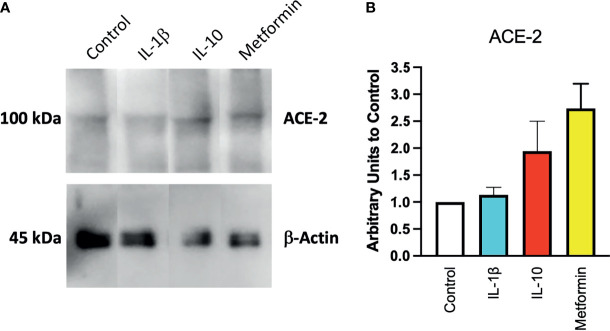
Expression of ACE2 protein in Calu-3 cells. ACE2 expression on Calu-3 cells was evaluated following 24-h exposure to IL-1β (25 ng/ml) and IL-10 (5 and 25 ng/ml) and metformin (10 mM) by Western blot **(A)**. Band intensities were denistometrically quantified **(B)** and normalized to β-actin and control; *N* = 4. Data are shown as mean ± SEM. Control, control vehicle cells.

To confirm at the protein level the presence of ACE2, ACE2 protein was detected by Western blot in Calu-3 cells (**Figure 1A**) with specific antibodies. Notwithstanding the constitutive presence of ACE2 on the membrane of these cells, modulation at the protein level was observed following 24-h treatment of IL-10 (**Figure 2A**). We found elevated ACE2 expression in Calu 3 cells treated with IL-10 (25 ng/ml) but not IL-1β (**Figure 2A**). Metformin treatment also increased ACE2. Data of four independent Western blot were quantified and reported in a histogram (**Figure 2B**).

### IL-10 Treatment of HUVE (Endothelial) Cells

**Figure 3:**
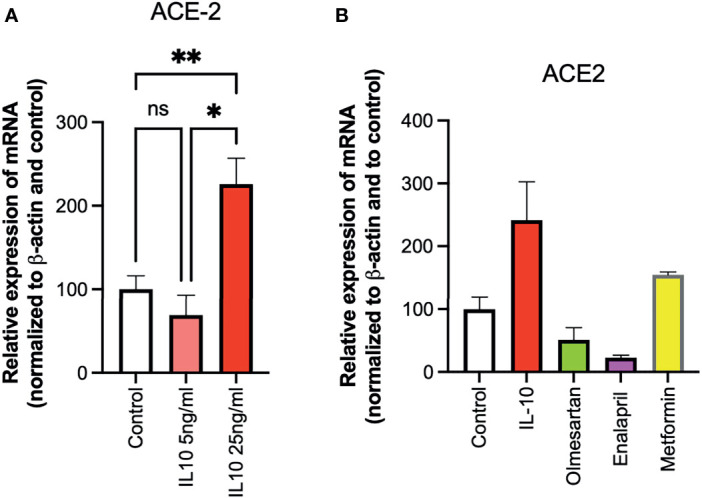
Effects of IL-10, sartans, and metformin on ACE2 expression in HUVECs. The ability of IL-10 (5 and 25 ng/ml) to induce ACE2 expression in HUVECs was determined, following 24 h of stimulation, by qPCR. **(A)** IL-10 increased ACE2 expression in a dose-dependent manner. **(B)** The effects of IL-10 (25 ng/ml) on ACE2 expression in HUVECs was determined by qPCR, olmesartan (10 µM), enalapril (10 µM), or metformin (10 mM). Metformin shows a trend in increasing ACE2 expression in HUVECs. Data are shown as mRNA relative expression, normalized to β-actin and control, mean ± SEM, one-way ANOVA, ^*^ *p* < 0.05, ^**^ *p* < 0.01. Control, control vehicle cells. ns, not significant.

The ability of IL-10 (5 and 25 ng/ml) to induce ACE2 expression in HUVECs was determined, following 24 h of stimulation, by qPCR. IL-10 increased ACE2 expression and was statistically significantly higher upon treatment with IL-10 at 25 ng/ml (**Figure 3A**). The effects of IL-10 (25 ng/ml) on ACE2 expression in HUVECs was compared to those of olmesartan (angiotensin II receptor inhibitors) and enalapril (ACE inhibitor), by qPCR. Olmesartan and enalapril did not enhance expression, they even induced a small but not significant decrease in ACE2 (**Figure 3B**). Cells treated with the biguanide metformin exhibited significantly upregulation of ACE2 (**Figure 3B**).

**Figure 4:**
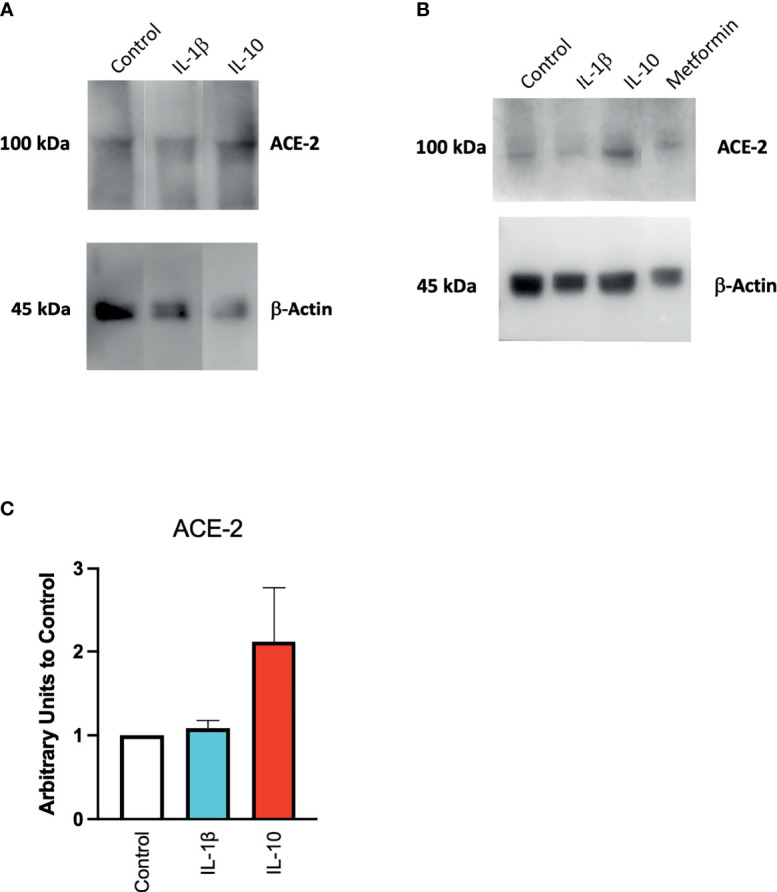
Expression of ACE2 protein in HUVECs. ACE2 expression by HUVECs was evaluated following 24-h exposure to IL-1β (25 ng/ml) or IL-10 (25 ng/ml) **(A)**, IL-10 (5 ng/ml), IL-1β (25 ng/ml) and metformin (10 mM) **(B)** by Western blot. Eight Western blots were quantified, normalized to β-actin and control, and reported in a histogram **(C)**.

We also found a modulation in ACE2 protein expression in HUVECs treated with IL-10 (25 ng/ml) by Western blot (**Figure 4**) stained with specific antibodies, as compared to cytokine IL-1β treatment and control untreated cells. We observed that IL-10 (25 ng/ml) increased ACE2 in HUVECs, while IL-1β decreased it (**Figure 4A**). Also, 5 ng/ml of L-10 were able to show induction of ACE2 protein (**Figure 4B**). Bands of eight Western blots were scanned, quantified, and reported in graphic (**Figure 4C**). HUVECs treated with metformin also exhibited upregulation of ACE2 (**Figure 4B**).

## Discussion

Ang-II stimulates the expression of pro-inflammatory mediators, including IL-6, through receptor subtypes (20). Ang II can be hydrolyzed from ACE2 to Ang1–7, which can bind to MAS [G protein-coupled receptor (GPCR)] receptors to act as an antagonist of Ang-II receptors to mediate physiological processes (20). An increase in the activation of ACE2, which produces Ang-1-7 that binds to MAS receptors, prevents local and systemic dysfunctions and reduces inflammation (20, 52); it is a shared opinion that ACE2 plays a protective role in acute lung injury (20, 52). A reduced expression of ACE2 caused by SARS-CoV-2 infection could contribute the SARS inflammatory effect (53). In SARS-CoV2 infection, ACE2 plays a dual role. On the one hand, it is the principal SARS-CoV-2 receptor and previous studies have interrelated the susceptibility of human airway epithelia to SARS-CoV-2 infection through the presence of ACE2 (15–17). On the other hand, the expression of mRNA and the enzymatic activity of ACE2 can decrease the inflammatory activity and exert a protective role in acute lung injury (20). In a rat model, resorcinolnaphthalein, which increases the activity of ACE2, improved endothelia-dependent vasorelaxation and increased the anti-inflammatory cytokine IL-10 (54, 55).

A recent *Nature Medicine* paper shows that manipulation of RAAS and supraphysiological levels of ANGII in swine induce a pathological phenotype that shares several features of COVID-19 (56). In swine, blocking ACE2 leads to increased coagulation, disturbs lung perfusion, induces diffuse alveolar damage, increases pulmonary artery pressure, and reduces blood oxygenation differently compared to control animals (56).

A decrease in ACE2 can be involved in ARDS. An increased and exacerbated immune response has been associated with the high virulence of SARS-CoV (8, 9), indicated as “cytokine storm”. Peripheral blood and lungs of SARS patients are characterized by elevated levels of proinflammatory cytokines, such as IL-1, IL-2, IL-6, IL-8/CXCL8, and chemokines associated with disease severity (10–14).

Several reports show in particular that high serum levels of not only of the pro-inflammatory IL-6, IL-1β and TNFα but also anti-inflammatory cytokine IL-10 are associated with the severity of the disease and a higher comorbidity index among adults with COVID-19 (38, 39, 41–46, 57). Changes in serum IL-6 and IL-10 levels act as a predictive biomarker to determining severe patient COVID-19 (44).

Two meta-analyses of IL-6 and IL-10 circulating levels found a correlation between the disease severity and mortality in COVID-19 patients (45, 46). Since IL-10 is reported as an immune-modulating cytokine, elevated levels could represent a reaction of the organism to curb inflammation, a sort of alarmin-like signal (47).

Our working hypothesis was that the IL-10 action could be mediated by regulation of ACE2 receptor expression. Our preliminary data, which could have implication in fostering novel studies in this direction, show that IL-10 increases ACE2 mRNA expression in lung-derived Calu-3 cells, and this could be involved in ARDS regulation. Recently, it has been shown that a simple natural IL-10-inducing small molecule can ameliorate a chronic inflammatory disease (58).

A high frequency of thrombosis and thromboembolism has been additionally reported in COVID-19-affected patients [16–18] (**Figure 1**). ACE2 expression has been demonstrated in endothelial cells from arterial and venous vessels [5], and there is clear-cut evidence that endothelial cells can acquire SARS-CoV-2 infection [19], with development of endotheliitis, endothelial cell damage, systemic vasculitis, and disseminated intravascular coagulation (DIC). Our group has reported a case of biventricular thrombosis in a COVID-19 patient with ischemic dilated cardiomyopathy (59).

Here, we present preliminary evidence that anti-inflammatory cytokine IL-10 treatment induces ACE2 mRNA enhancement in endothelial cells. Regulation is opposite of the one induced by pro-inflammatory signal IL-1β. The sartans (olmesartan and losartan) have no significant modulation over the control groups.

Another molecule that has been suggested to be able to mitigate COVID-19 inflammatory syndrome is metformin (50, 51). A few retrospective analyses of observational studies in COVID-19 patients with type 2 diabetes mellitus, with and without metformin, have shown a reduction in mortality for metformin (50, 51). In a retrospective US large cohort, metformin was significantly linked to reduced death in women (51). A small Chinese cohort also examined metformin effects (56). Metformin has hypothetically been suggested for the treatment of patients with COVID-19 at risk of developing severe illness (60). In our report, metformin appears to act in the same direction as IL-10, by enhancing ACE2 mRNA expression. Metformin activates the NAD+-dependent deacetylase silent information regulator T1 (SIRT1), which regulates expression of ACE2 (61, 62) and cardiopulmonary protection in COVID-19 (62–64).

An early induction of anti-inflammatory mediator IL-10 upon SARS-CoV-2 infection might function as a mediator that serves as a countermeasure to inflammation and coagulopathy (40); given the dual IL-10 (32, 35) caution is mandatory. The initial evidence is limited, and further studies are warranted to confirm the role IL-10 in COVID-19.

## Conclusions

Treatments that increase ACE2 may be beneficial in mitigating the complications of COVID-19 by curbing inflammation. Our evidence shows that IL-10 is upregulating ACE2 in lung-derived cells and endothelial cells. We believe that IL-10, by enhancing ACE2, could be a body attempt to reduce inflammation. Although more investigations are required, it could be hypothesized that treatment with agents increasing IL-10, by reinforcing ACE2 expression or production of Ang 1–7 peptide, may represent a novel way to treat COVID-19-associated ARDS.

## Data Availability Statement

The original contributions presented in the study are included in the article/**Supplementary Material**. Further inquiries can be directed to the corresponding authors.

## Author Contributions

AA developed the hypothesis, designed the experiments, cultured cells, and wrote the manuscript. LC and VC performed the main experiments on Calu3 and HUVEC cells. NB performed additional experiments. ML discussed and wrote the clinical relevance of the results obtained. AB analyzed data, drafted protocols, and contributed to the manuscript writing. DN outlined and organized the studies and wrote the manuscript. All authors contributed to the article and approved the submitted version.

## Funding

This research was funded by a grant from the Ministero della Salute COVID-2020-12371849 to DMN. Studies are partially supported by the Italian Ministry of Health Ricerca Corrente—IRCCS MultiMedica. AB has received funds from the Italian Association for Cancer Research (AIRC-MFAG id-22818) and the Cariplo Foundation (id- 2019-1609).

## Conflict of Interest

The authors declare that the research was conducted in the absence of any commercial or financial relationships that could be construed as a potential conflict of interest.

## Publisher’s Note

All claims expressed in this article are solely those of the authors and do not necessarily represent those of their affiliated organizations, or those of the publisher, the editors and the reviewers. Any product that may be evaluated in this article, or claim that may be made by its manufacturer, is not guaranteed or endorsed by the publisher.
