# Supplementary material for: Preliminary Evidence for IL-10-Induced ACE2 mRNA Expression in Lung-Derived and Endothelial Cells: Implications for SARS-Cov-2 ARDS Pathogenesis
Source: Front Immunol. 2021 Sep 27;12:718136. doi: 10.3389/fimmu.2021.718136 (PMC8503675; doi:10.3389/fimmu.2021.718136)
Supplement: Supplementary file 1 [file Table_1.pdf]

Supplemental table 1

Primers	
ACE-2	
FWD-1	5'-TCCATTGGTCTTCTGTCACCCG-3'
REV-1	5'-AGACCATCCACCTCCACTTCTC-3'
FWD-2	5'-AGAAAGCAGTCTGCCATCCC-3'
REV-2	5'-AGTCGGTACTCCATCCCACA-3'
β-actin	
FWD-1	5'-CACCATTGGCAATGAGCGGTTC-3'
REV-1	5'-AGGTCTTTGCGGATGTCCACGT-3'
FWD-2	5'-CACCATGTACCCTGGCATTG-3'
REV-2	5'-GGCTTGCTGATCCACATCTG-3'
